# The Cortical States of Wakefulness

**DOI:** 10.3389/fnsys.2018.00064

**Published:** 2019-01-08

**Authors:** James F. A. Poulet, Sylvain Crochet

**Affiliations:** ^1^Neural Circuits and Behaviour, Department of Neuroscience, Max Delbrück Center for Molecular Medicine (MDC), Berlin, Germany; ^2^Neuroscience Research Center and Cluster of Excellence NeuroCure, Charité-Universitätsmedizin Berlin, Berlin, Germany; ^3^Laboratory of Sensory Processing, Brain Mind Institute, Faculty of Life Sciences, École Polytechnique Fédérale de Lausanne (EPFL), Lausanne, Switzerland; ^4^Lyon Neuroscience Research Center, INSERM U1028/CNRS UMR5292, University Lyon 1, Lyon, France

**Keywords:** brain states, barrel cortex, sensory processing, synchrony, acetylcholine

## Abstract

Cortical neurons process information on a background of spontaneous, ongoing activity with distinct spatiotemporal profiles defining different cortical states. During wakefulness, cortical states alter constantly in relation to behavioral context, attentional level or general motor activity. In this review article, we will discuss our current understanding of cortical states in awake rodents, how they are controlled, their impact on sensory processing, and highlight areas for future research. A common observation in awake rodents is the rapid change in spontaneous cortical activity from high-amplitude, low-frequency (LF) fluctuations, when animals are quiet, to faster and smaller fluctuations when animals are active. This transition is typically thought of as a change in global brain state but recent work has shown variation in cortical states across regions, indicating the presence of a fine spatial scale control system. In sensory areas, the cortical state change is mediated by at least two convergent inputs, one from the thalamus and the other from cholinergic inputs in the basal forebrain. Cortical states have a major impact on the balance of activity between specific subtypes of neurons, on the synchronization between nearby neurons, as well as the functional coupling between distant cortical areas. This reorganization of the activity of cortical networks strongly affects sensory processing. Thus cortical states provide a dynamic control system for the moment-by-moment regulation of cortical processing.

## Introduction

Even in the absence of any external sensory input or motor activity, the brain is constantly active. This ongoing, or spontaneous, electrical activity was first revealed in living animals by Caton (1875) and then by Berger (1929) in humans using electroencephalography (EEG) recordings on the skull surface. Hans Berger’s classical EEG recordings from the occipital cortex of awake, but relaxed, subjects with their eyes closed showed high-amplitude oscillatory activity around 10–15 Hz that transitioned rapidly to smaller and faster fluctuations whenever the subject opened his eyes or performed mental calculations. This pioneering work was the first report of a brain state change correlated with a change in behavioral or mental state. It also introduced the notion of brain rhythms or oscillations, and the idea that different brain states could be characterized by the dominant frequency component of the EEG activity.

Following these early studies, the use of scalp EEG has been extensively used to study cortical activity in relation to behavioral states, neurological disease and mental processes in humans and animal models. Many studies have characterized cortical activities across the wake-sleep cycle (Loomis et al., 1935; Rheinberger and Jasper, 1937; Moruzzi and Magoun, 1949; Jouvet, 1967; Steriade et al., 1993b; Hobson and Pace-Schott, 2002), reporting small-amplitude high-frequency (HF) fluctuations of the EEG during wakefulness that progressively transition to higher-amplitude and slower fluctuations when the subject falls asleep to reach maximum amplitude and lowest frequency during non-rapid-eye-movement (non-REM) sleep. Because EEG recordings during non-REM sleep are dominated by slow patterns of electrical activity (the “slow-oscillation” 0.1–1.5 Hz and delta activity 1.5–5 Hz), this sleep state is also referred to as slow-wave sleep (SWS)^1^. Based on these early EEG studies, wakefulness is often described as a state of global neocortical *desynchronization*, dominated by low-voltage, HF (>20 Hz) activities, whereas NREM sleep is seen as a state of global *synchronization* dominated by high-voltage, low-frequency (LF, <10 Hz) activities (Lin, 2000; Steriade, 2000; Hobson and Pace-Schott, 2002; Jones, 2005; Brown et al., 2012). However, later studies have demonstrated that HF cortical activities, in particular in the gamma frequency range (30–90 Hz), can be highly synchronous during wakefulness within and across cortical areas (Steriade et al., 1996; Destexhe et al., 1999; Steriade, 2000; Engel et al., 2001). Consequently, the terms *activated* and *deactivated* were proposed to replace the terms *desynchronized* and *synchronized* respectively. In this review article, we will refer to *activated state* or *cortical activation* for cortical activity characterized by a low ratio between LF (1–10 Hz) and HF (20–100 Hz) activity.

Until recently, the cellular mechanisms underlying cortical states in awake mammals was poorly understood due to technical limitations associated with manipulating and recording from identified neurons in awake animals. The development of the head-restrained mouse preparation made it possible to use a variety of electrophysiological and imaging techniques with cellular resolution in awake and behaving mice (Margrie et al., 2002; Petersen et al., 2003; Crochet and Petersen, 2006; Crochet, 2012). The combination of these techniques with genetically modified mouse lines, viral approaches and optogenetic tools have begun to shed new light on cortical activities and their correlation to behavioral states. Perhaps the most robust and apparent feature of cortical activity in awake mice has been the dramatic change in cortical activity when mice transition from quiet, immobile wakefulness, to an active motor behavior. In this article, we review our current understanding of the cellular mechanisms underlying this state change in mice, at both local and global levels, and the functional consequence of this state change on cortical processing, with special emphasis on the whisker primary somatosensory cortex.

## Brain State Change in the Barrel Cortex

**Figure 1:**
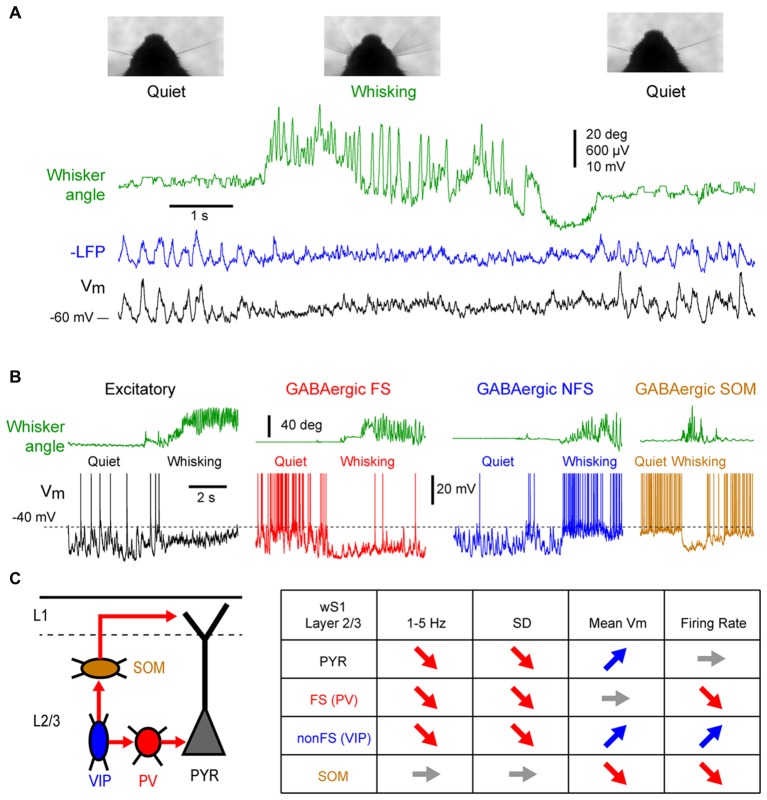
State change during whisking in the mouse barrel cortex.** (A)** Example simultaneous recording of the membrane potential (Vm) of a layer 2/3 pyramidal neuron (Black trace) and local field potential (LFP; Blue trace, shows reversed polarity). The green trace shows the angular position of the contralateral whisker extracted from high-speed video filming (Top images). Adapted from Poulet and Petersen (2008) with permission from Springer Nature. **(B)** Example Vm recordings from layer 2/3 neurons in the barrel cortex of awake mice during transition from quiet wakefulness (QW) to whisking (whisker angle, green). From left to right: an excitatory neuron (black); a fast-spiking (FS) GABAergic interneuron (red); a non-FS (NFS) GABAergic interneuron (blue); and a somatostatin (SOM) expressing GABAergic interneuron (orange). Adapted from Gentet et al. (2010) with permission from Elsevier and Gentet et al. (2012) with permission from Springer Nature. **(C)** *Left*, schematic representation of a simplified local circuit in layer 2/3 barrel cortex. Vasointestinal peptide (VIP) expressing interneurons inhibit parvalbumin (PV) and SOM expressing interneurons. PV interneurons provide perisomatic inhibition onto excitatory pyramidal (PYR) cells, whereas SOM interneurons target preferentially their apical dendrites in layer 1. *Right*, table summarizing the main effects of the transition from quiet to active wakefulness (AW) on different cell types of the layer 2/3 in the barrel cortex: 1–5 Hz Vm fluctuations; Vm standard deviation (SD); mean Vm; and mean firing rate.

In sharp contrast with earlier membrane potential (Vm) recordings using sharp electrodes in awake head-restrained cats (Steriade et al., 2001; Timofeev et al., 2001), whole-cell patch-clamp recordings in head-restrained mice during quiet wakefulness (QW) have reported pronounced LF fluctuations of the Vm of pyramidal cells in the whisker primary somatosensory (*barrel*) cortex (wS1; Petersen et al., 2003). Correlating Vm recording with behavioral quantification of the mouse motor activity (the movement of a whisker) revealed two distinct states in the mouse barrel cortex: during quiescent states, in the absence of whisker movements, large amplitude (10–20 mV) LF (<10 Hz) fluctuations dominates the Vm of layer 2/3 pyramidal cells, whereas during active whisker movements (whisking), the large and slow Vm fluctuations are replaced by smaller amplitude and faster fluctuations (Figure 1A); the pyramidal neurons depolarize slightly, without an overall change in firing rate at the population level (some neurons increase while others decrease their firing rate; Crochet and Petersen, 2006; Poulet and Petersen, 2008; de Kock and Sakmann, 2009; Crochet et al., 2011; Reimer et al., 2014).

While the first whole-cell recordings were performed blindly allowing only for *post hoc* cell identification, the combination of whole-cell recordings with *in vivo* two-photon microscopy made it possible to target recordings to specific cell populations identified either genetically using fluorescent protein expression, or by their projection targets using retrograde fluorescent labeling (Margrie et al., 2003; Komai et al., 2006). These approaches have shown cell type specific changes in activity during different cortical states. For example, among the layer 2/3 pyramidal neurons, those projecting to the whisker primary motor cortex (wM1) exhibit larger slow Vm fluctuations during QW and therefore a more pronounced change in subthreshold activity during transition from quiet to active wakefulness (AW) compared to the neurons that project to the secondary somatosensory cortex (wS2; Yamashita et al., 2013). The activity of genetically identified GABAergic interneurons in the layer 2/3 of the barrel cortex shows a profound reorganization during state change (Figures 1B,C). Fast-spiking (FS) interneurons [presumably expressing parvalbumin (PV)], as well as non-fast spiking interneurons (presumably expressing the 5HT3R serotonergic receptor) show large and slow Vm fluctuations during QW, and pronounced state change during AW, with a strong decrease in the slow fluctuations of the Vm (Gentet et al., 2010). The FS interneurons fire at high frequency during QW and decrease their firing rates during AW, whereas the non-fast spiking interneurons fire at lower rate during QW but depolarize and increase firing rate during active behavior (Gentet et al., 2010). An important subclass of non-fast spiking interneurons, expressing vasointestinal peptide (VIP; Gentet, 2012; Tremblay et al., 2016), have been found to markedly increase activity during locomotor activity in the mouse barrel cortex (Lee et al., 2013; Muñoz et al., 2017). In contrast to the other interneuron subtypes, the Layer 2/3 somatostatin (SOM) expressing interneurons display only small slow Vm fluctuations during QW that tend to be out of phase with the other cortical neurons (Gentet et al., 2012; Pala and Petersen, 2018). During AW, they show little change in the slow Vm fluctuations but they hyperpolarize and stop firing (Gentet et al., 2012; Lee et al., 2013; Muñoz et al., 2017; Pala and Petersen, 2018). The hyperpolarization of layer 2/3 SOM interneurons during active behaviors most likely originates from GABAergic inputs from VIP interneurons (Lee et al., 2013; Pfeffer et al., 2013; Pi et al., 2013; Muñoz et al., 2017) and can lead to a disinhibition of pyramidal neuron apical dendrites (Figure 1C; Gentet et al., 2012). While less is known about cell-type specific activity in deeper cortical layers, the modulation of neuronal activity by locomotor activity appears to vary substantially across layers. In layers 4 and 5, AW is associated with an overall increase in spike rate in excitatory pyramidal neurons (de Kock and Sakmann, 2009; Yu et al., 2016). In contrast to L2/3, SOM and FS (presumably PV) interneurons in layer 4 increase their activity during active movements, and show both increase and decrease in deeper layers (Yu et al., 2016; Muñoz et al., 2017). Thus, the transition between quiet and AW is accompanied by a rapid transition in the activity of the somatosensory cortex. The most striking effect is a strong reduction of the LF, high-amplitude fluctuations of Vm that results in a strong reduction of the variability of the subthreshold neuronal activity. In addition, a major functional reorganization of the inhibitory interneuron network takes place that may strongly impact cortical processing.

## Brain States in Other Cortical Areas

**Figure 2:**
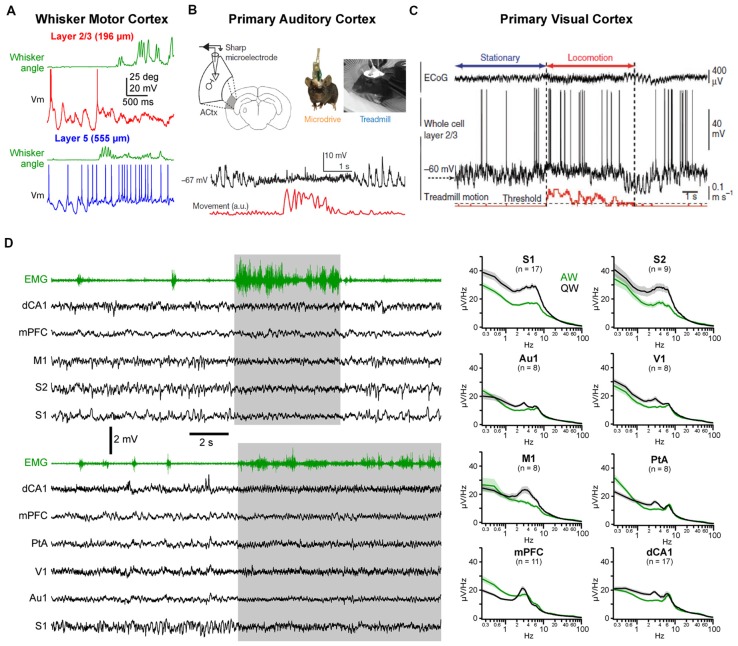
State change during AW across cortical areas.** (A)** State change during whisking (whisker angular position in green) observed in a layer 2/3 (top, red) and in a layer 5 (bottom, blue) pyramidal neuron recorded in the whisker primary motor cortex (M1) of awake head-fixed mice. Adapted from Sreenivasan et al. (2016) with permission from Elsevier.** (B)** Vm recording in the Au1 of a freely moving mouse reveal state change during locomotor activity (movement, red). From Schneider et al. (2014) with permission from Springer Nature. **(C)** Vm recording in the primary visual cortex (V1) of a head-fixed mouse shows similar state change during locomotion. From Polack et al. (2013) with permission from Springer Nature. **(D)** *Left*, examples multisite LFP recordings during QW and AW in head-fixed mice. The nuchal electromyogram (EMG, green traces) is used to monitor the overall motor activity. In the top example, LFPs were recorded from the dorsal CA1 region of the hippocampus (dCA1), the medial prefrontal cortex (mPFC), the M1, the secondary (S2) and primary (S1) somatosensory cortices. In the bottom example, LFPs were recorded from dCA1, mPFC, the parietal associative area (PtA), the V1, auditory (Au1) and somatosensory (S1) cortices. *Right*, the spectral analysis of the LFPs shows a general decrease in LF (1–10 Hz) activity during AW (green) compared to QW (black). Adapted from Fernandez et al. (2017) with permission from Oxford University Press.

A similar change in activity during the transition from quiet to active behaviors has also been reported in other cortical areas. In the forepaw region of S1, both supragranular (layer 2/3) and infragranular (layer 5) pyramidal cells show a strong suppression of LF Vm fluctuations and a depolarization of the mean Vm, but only infragranular cells increase their firing rates (Zhao et al., 2016). In the wM1, an area strongly synaptically interconnected with wS1 (Aronoff et al., 2010; Mao et al., 2011; Kinnischtzke et al., 2014; Zingg et al., 2014; Sreenivasan et al., 2016; Yamashita et al., 2018), a transition from large and slow fluctuations to small and fast fluctuations has been observed both with local field potential (LFP) and Vm recordings (Figure 2A; Zagha et al., 2013; Sreenivasan et al., 2016; Fernandez et al., 2017). Interestingly, the mean firing rate of excitatory neurons shows only a transient increase in infragranular layers, and a suppression in superficial layers (Sreenivasan et al., 2016). In freely moving rats, extracellular recordings from infragranular layers have shown an overall, weak, reduction of the mean spike rate of wM1 neurons during whisking, with only a minority of neurons increasing activity (Ebbesen et al., 2017). In forelimb M1, similar changes in cortical state are observed as mice go from stationary to running that strongly affect the subthreshold activity in both L2/3 and L5—with a pronounced decrease in LF Vm fluctuations—but have various effects on the firing rate—with both enhanced and suppressed neurons intermingled in L5A and B and almost no effect on the spike rates of L2/3 neurons (Schiemann et al., 2015). Thus, somatosensory and associated motor areas show similar overall patterns of state change (Figure 2D; Zagha et al., 2013; Fernandez et al., 2017).

Changes in spontaneous cortical activity correlated with behavior has also been reported in other sensory cortices, including primary auditory cortex (Au1) and primary visual cortex (V1; Figures 2B–D). A common feature of the state change across cortical areas is the decrease in LF Vm fluctuations during movement resulting in a reduced variance [or standard deviation (SD)] of the Vm in pyramidal neurons (Bennett et al., 2013; Polack et al., 2013; Reimer et al., 2014; Schneider et al., 2014; Zhou et al., 2014; Neske and McCormick, 2018). Excitatory neurons in V1 tend to depolarize during AW (Bennett et al., 2013; Polack et al., 2013; Reimer et al., 2014; Neske and McCormick, 2018) but various effects have been reported on firing rate (Bennett et al., 2013; Polack et al., 2013; Erisken et al., 2014; Vinck et al., 2015; Dipoppa et al., 2018; Neske and McCormick, 2018). In Au1, both a net depolarization and a net hyperpolarization of layer 2/3 excitatory neurons have been found but accompanied overall by a decrease in firing rate^2^ (Schneider et al., 2014; Zhou et al., 2014). As in wS1, VIP interneurons in V1 depolarize and show an increased firing rate during locomotion (Fu et al., 2014; Reimer et al., 2014; Pakan et al., 2016; Dipoppa et al., 2018). Variable effects on the mean Vm and spike rates have been observed for PV and SOM interneurons in V1 (Polack et al., 2013; Fu et al., 2014; Vinck et al., 2015; Pakan et al., 2016; Dipoppa et al., 2018) and Au1 (Schneider et al., 2014; Zhou et al., 2014). Part of these discrepancies might be ascribed to layer specificities (Dipoppa et al., 2018) or behavioral context (Pakan et al., 2016). Altogether, it appears that locomotor activity drives a global state change across widespread cortical areas (Fernandez et al., 2017; Shimaoka et al., 2018). In most cortical areas, this state change is associated with a marked decrease in LF, large amplitude subthreshold activity whereas HF activities are preserved or increased, resulting in a global cortical activation. However, because LF activity during QW is more pronounced in somatosensory and motor areas, the contrast in cortical activity observed between quiet and AW is especially clear in those areas (Figure 2D; Fernandez et al., 2017).

## Multiple Brain States During Wakefulness

As we have described, splitting datasets into QW or active motor behavior leads to a clear difference in the dynamics of neuronal activity across the rodent cortex. However, this simple classification does not fully represent the richness and complexity of the cortical activity during wakefulness. The intensity and type of movement—with different end goals and sensory feedback—may have a different impact on cortical activity. A recent study, for example, compared changes in neuronal activity of pyramidal neurons in L2/3 and L5 of wS1 as mice are immobile, during whisker movements and during locomotion (which is always accompanied by whisking). In agreement with previous studies, whisking alone had only minor impact on the mean activity of pyramidal neurons, whereas running was accompanied by an increased activity in the majority of the neurons (Ayaz et al., 2018). Thus, locomotion has an additional impact on cortical activity compared to whisking alone. Future studies should investigate whether different types of skilled movement such as reaching, or free exploration of natural environments may also lead to behavior-specific changes of cortical state.

**Figure 3:**
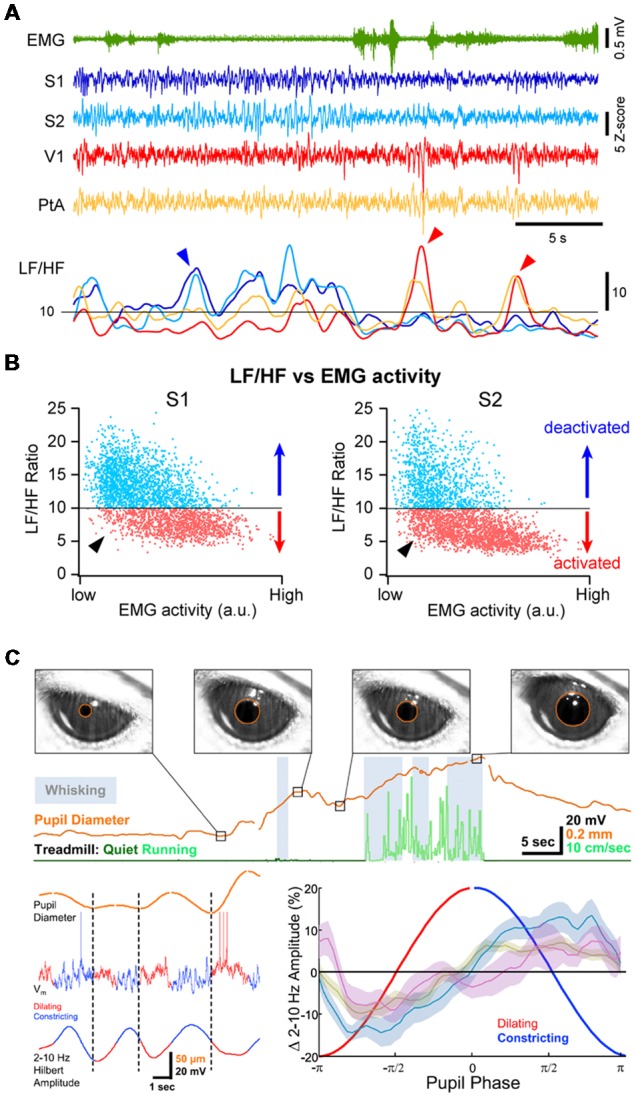
Multiple cortical states during wakefulness.** (A)** Cortical activation/deactivation can occur independently across cortical areas. Example simultaneous LFP recordings (z-scored) from S1, S2, V1 and PtA. The ratio between the low-frequency (LF; 1–10 Hz) and high-frequency (HF; 30–90 Hz) activity of the LFP can be used to assess the level of cortical activation. The LF/HF ratio from the depicted LFPs (Bottom) indicates periods of deactivation in S1 and S2 while PtA and V1 are in an activated state (blue arrowhead) and periods of deactivation in V1 and PtA while S1 and S2 are activated (red arrow heads). Adapted from Fernandez et al. (2017) with permission from Oxford University Press. **(B)** Plotting the LF/HF ratio against the motor activity (EMG) reveals cortical state fluctuations during QW (low EMG activity). Examples from recordings in the primary (S1, *Left*) and secondary (S2, *Right*) somatosensory cortices of an awake mouse. While cortical activation largely dominates during high motor activity, both deactivated and activated (arrowhead) states can be observed during periods of QW. Adapted from Fernandez et al. (2017) with permission from Oxford University Press. **(C)** Pupil diameter fluctuates during wakefulness. *Top*, Pupils dilate during locomotion but pupil dilations are also observed during QW in the absence of locomotor activity. *Bottom*, Pupil dilation is associated to cortical activation. *Left*, example Vm recording in the V1 of an awake mouse together with the monitoring of the pupil diameter (top trace). The amplitude of the LF Vm fluctuations is shown below (2–10 Hz Hilbert amplitude). *Right*, mean amplitude of the LF Vm fluctuations plotted as function to the phase of the pupil diameter. Note the low amplitude of the LF fluctuations during the dilating phase compared to the constricting phase. Olive, recordings from wS1; blue, recordings from V1; mauve, recordings from V1 in FVB mice. Adapted from Reimer et al. (2014) with permission from Elsevier.

Periods of cortical activation are also observed in the absence of any overt behavior. This can be revealed by plotting the ratio of LF over HF activity (LF/HF ratio) against the motor activity: during high motor activity, the cortex is always in an activated state (i.e., low LF/HF ratio) but during QW, cortical states fluctuate between an activated and a deactivated state (Figures 3A,B; Urbain et al., 2015; Fernandez et al., 2017). Recent studies have pointed to a close correlation between transient active cortical states, pupil diameter and attention or arousal. Pupils dilate during locomotor activity (Erisken et al., 2014; Reimer et al., 2014; Vinck et al., 2015; Shimaoka et al., 2018), but also fluctuate during quiet, immobile wakefulness (Figure 3C; Reimer et al., 2014; McGinley et al., 2015a; Vinck et al., 2015). Pupil dilation is associated with a decrease in LF Vm fluctuations in wS1 and V1, independent from locomotor activity (Figure 3C; Reimer et al., 2014), while in Au1, pupil dilation in the absence of locomotion is associated with Vm depolarization (McGinley et al., 2015a). Pupil diameter fluctuations are believed to reflect change in the activity of the noradrenergic neurons in the locus coeruleus (Joshi et al., 2016; Binda and Gamlin, 2017) and are correlated with an increased activity in cholinergic and noradrenergic axons in sensory cortices (V1 and Au1; Reimer et al., 2016). Thus, pupil dilation is well correlated to periods of cortical activation in the absence of motor activity that could reflect arousal or attention driven by ascending neuromodulators and distinct from locomotor activity.

Cortical activity is often seen to fluctuate between different states at a cortex wide scale. Recent studies investigating the fluctuations in neuronal activity across cortical regions in awake mice during spontaneous behaviors or execution of a sensory decision-making task, have found that most of the brain-wide fluctuations could be explained by arousal and motor activity (Musall et al., 2018; Stringer et al., 2018). However, simultaneous recordings of cortical activities from several cortical areas using multisite LFP recordings in awake mice or rats have also revealed that cortical activation or deactivation can occur locally, especially during QW (Vyazovskiy et al., 2011; Fernandez et al., 2017). For instance, short periods of high-amplitude, LF activity can be recorded in V1 while wS1 is activated and vice-versa (Figure 3A). Correlating the cortical state (LF/HF) across areas revealed a functional organization, with highly correlated state fluctuations in functionally-linked cortical regions (e.g somatosensory and motor or visual, auditory and parietal areas; Fernandez et al., 2017). Thus, although cortical states can be globally regulated reflecting overall arousal or locomotor activity, finer, more local, state regulation occurs potentially reflecting attentional shifts to different sensory or motor systems. Interestingly, similar local regulation of cortical activity has been observed during non-REM sleep (Huber et al., 2004; Nir et al., 2011).

## Cellular Mechanisms Controlling Cortical States

**Figure 4:**
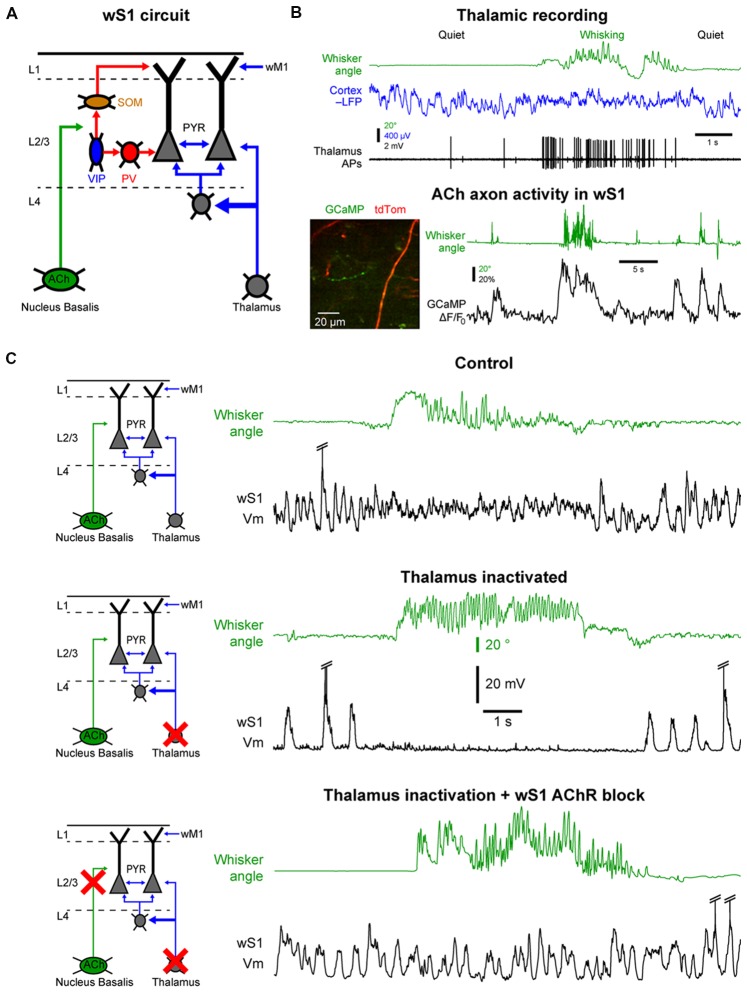
Cellular mechanisms of the state change in the barrel cortex.** (A)** The whisker primary somatosensory cortex (wS1) receives three main inputs coming from the thalamus, the cholinergic neurons in the basal forebrain and the whisker M1 (wM1). **(B)** Thalamic and cholinergic inputs increase activity during whisking. *Top*, example single-unit recording from a thalamic neuron (Thalamus APs, black) together with LFP recording in wS1 (Cortex LFP, blue, reversed polarity) and monitoring of the whisker position (Whisker angle, green). Adapted from Poulet et al. (2012) with permission from Springer Nature. *Bottom*, example 2-photon calcium imaging (GCaMP) of cholinergic axons in wS1. Adapted from Eggermann et al. (2014) with permission from Elsevier. **(C)** Both thalamic and cholinergic inputs contribute to the state change in wS1 during whisking. *Top*, example control recording of Vm in wS1 during quiet and whisking periods. *Middle*, example recording in wS1 following pharmacological inactivation of the thalamus. *Bottom*, example recording in wS1 following inactivation of the thalamus and local blockade of the cholinergic receptors. Note that the simultaneous blockade of the thalamic and cholinergic inputs abolishes the state change during whisking in wS1. Top and middle panels are adapted from Poulet et al. (2012) with permission from Springer Nature; Bottom panel is adapted from Eggermann et al. (2014) with permission from Elsevier.

Cortical neurons are primarily innervated by other local cortical neurons and it is established that cortico-cortical connectivity is critical for the generation of slow spontaneous cortical activity during sleep or anesthesia (Sanchez-Vives and McCormick, 2000; Timofeev et al., 2000; Beltramo et al., 2013). However, direct inputs from other cortical areas or subcortical structures also contribute markedly to the generation and modulation of cortical activities and in this section, we will discuss the relative contribution of three main external inputs to the barrel cortex in controlling the state change associated to locomotor activity: (1) glutamatergic inputs from the thalamus; (2) cholinergic inputs from the basal forebrain; and (3) top-down glutamatergic inputs from motor cortex (Figure 4A). Finally, we examine the control of cortical states by brainstem nuclei that do not directly project to neocortex.

One of the densest external, non-cortical, innervation of the barrel cortex comes from the thalamus. Thalamocortical neurons are glutamatergic and therefore provide an important excitatory drive to the cortex. Two major thalamic nuclei project to the barrel cortex, the ventral posteromedial nucleus (VPM) and the posteromedial nucleus (POm; Diamond et al., 2008; Wimmer et al., 2010; Bosman et al., 2011). The VPM relays sensory information from the vibrissa through the brainstem in a highly specific manner that conserves the spatial organization of the vibrissa. The POm also receives tactile sensory information via the brainstem but in addition, it receives broad and strong cortical inputs and single POm neurons innervate broad cortical regions. It is therefore considered to be a “higher-order” thalamic nucleus (Feldmeyer et al., 2013; Groh et al., 2014; Jouhanneau et al., 2014; Sherman, 2016). The activity of thalamocortical neurons in the VPM and POm is highly correlated to cortical activation (Figure 4B). These neurons are tonically active during wakefulness and increase their firing rate during motor activity (Poulet et al., 2012; Urbain et al., 2015; Yu et al., 2016). Interestingly, in the whisker system, the activity of VPM neurons seems to be more correlated to whisker movements, whereas POm neurons increase firing rates during cortical activation even in the absence of overt motor activity (Urbain et al., 2015). A blockage of thalamocortical inputs leads a pronounced increase in the slow Vm fluctuations in wS1 during QW, with clear Up and Down states resembling those observed under anesthesia. Surprisingly, in mice with thalamus inactivated, there is a complete shut-down of cortical activity in wS1 during AW, similar to a prolonged Down state (Figure 4C). Moreover, optogenetic activation of thalamocortical neurons leads to strong cortical activation in wS1 even in the absence of any whisker movements (Poulet et al., 2012). Together these data suggest that the thalamic excitatory drive is responsible for the depolarization and HF Vm activity in wS1 neurons during cortical activation. But what causes the prolonged Down states during movement after thalamic inactivation?

The barrel cortex is densely innervated by non-glutamatergic neuromodulatory systems releasing acetylcholine, noradrenaline, serotonin or histamine. These ascending neuromodulatory systems are more active during wakefulness and reduce or stop firing during sleep (Lin, 2000; Jones, 2005). Among them, the cholinergic system arising from the basal forebrain seems to play an important role in maintaining arousal and attention and has been heavily studied in the context of cortical activation during wakefulness (Buzsaki et al., 1988; Metherate et al., 1992; Pinto et al., 2013; Záborszky et al., 2018). Recently, functional calcium imaging showed an increase in cholinergic neuron activity during AW compared to QW (Figure 4B; Eggermann et al., 2014; Harrison et al., 2016). Moreover, optogenetic activation of cholinergic neurons during thalamic inactivation reproduces the state change observed during AW, i.e., the suppression of cortical activity. This effect is blocked by the local pharmacological blockage of cholinergic receptors in wS1. Simultaneous local blockage of cholinergic receptors in wS1 together with thalamic inactivation completely abolishes the state change observed in wS1 during AW (Figure 4C; Eggermann et al., 2014). A similar cholinergic suppression of cortical activity is also observed in anesthetized mice (Meir et al., 2018). Thus, in wS1, cholinergic inputs are responsible for the abolition of the slow Vm fluctuations, whereas thalamic inputs drive the depolarization and fast Vm fluctuations during AW.

Acetylcholine acts on cortical neurons through the activation of nicotinic and muscarinic receptors, both located at presynaptic and postsynaptic sites. Direct postsynaptic nicotinic excitatory responses have been reported in some deep layer pyramidal neurons in wS1 (Hedrick and Waters, 2015) as well as in VIP interneurons (Arroyo et al., 2012; Fu et al., 2014; Pronneke et al., 2018). Activation of nicotinic receptors can facilitate thalamocortical transmission (Gil et al., 1997; Oldford and Castro-Alamancos, 2003; Kruglikov and Rudy, 2008), therefore potentially increasing the impact of external sensory inputs. Nicotinic receptors has been shown recently to facilitate glutamatergic transmission between Pyramidal and SOM interneurons, potentially increasing the recruitment of SOM interneurons by pyramidal cells (Urban-Ciecko et al., 2018). Muscarinic receptors can produce both slow postsynaptic excitatory or inhibitory responses depending on the receptor subtype. For example, acetylcholine exerts an excitatory muscarinic effect onto SOM interneurons (Chen et al., 2015; Muñoz et al., 2017) and an inhibitory muscarinic effect onto L4 excitatory neurons (Eggermann and Feldmeyer, 2009; Dasgupta et al., 2018). Muscarinic receptors can also reduce cortico-cortical interactions by depressing glutamatergic cortico-cortical synaptic transmission (Gil et al., 1997; Hsieh et al., 2000; Eggermann and Feldmeyer, 2009). A decrease in leak or voltage-activated potassium conductances in pyramidal cells could also contribute to the suppression of the slow cortical activity by acetylcholine, although it would be expected to maintain cortical neurons in a prolonged Up state rather than in Down state (McCormick and Williamson, 1989; Sanchez-Vives and McCormick, 2000; Compte et al., 2003). Acetylcholine release during AW could therefore have multiple effects, leading to the disinhibition of the apical dendrites of pyramidal cells through nicotinic activation of VIP interneurons in superficial layers; leading to direct muscarinic inhibition of L4 cells and indirect inhibition of deep pyramidal neurons through muscarinic excitation of SOM interneurons; and suppressing cortico-cortical interaction through presynaptic muscarinic receptors while enhancing thalamocortical inputs through presynaptic nicotinic receptors (Gil et al., 1997; Disney et al., 2007). There is good evidence that recurrent slow cortical activities are cortically generated and rely strongly upon cortico-cortical synaptic interaction (Sanchez-Vives and McCormick, 2000; Timofeev et al., 2000; Poulet et al., 2012) as well as the activity of a subset of pyramidal neurons in infragranular layer (Sakata and Harris, 2009; Beltramo et al., 2013; Zhao et al., 2016). It is thus possible that the main action of acetylcholine in the state change is to abolish the slow spontaneous cortical activity by the depression of cortico-cortical synaptic transmission and/or inhibition of pyramidal neurons through the activation of muscarinic receptors (Eggermann and Feldmeyer, 2009; Favero et al., 2012; Eggermann et al., 2014; Dasgupta et al., 2018; Meir et al., 2018). The exact cellular and subcellular mechanisms of action of acetylcholine in the regulation of cortical states remain to be determined.

The barrel cortex receives also strong excitatory synaptic inputs from two other cortical areas, the secondary whisker somatosensory cortex (wS2) and the wM1 (Aronoff et al., 2010; Zingg et al., 2014). The wM1 is involved in the control of whisker movements (Haiss and Schwarz, 2005; Matyas et al., 2010; Hill et al., 2011; Sreenivasan et al., 2016; Ebbesen et al., 2017). It has been suggested that wM1 sends a copy of the descending motor command to wS1 and this signal could play a role in the state change in wS1 during whisker movements (Zagha et al., 2013). At the initiation of whisker movements, some L5 wM1 neurons increase activity and may increase further during locomotion or when engaged in a task (Petreanu et al., 2012; Sreenivasan et al., 2016). Optogenetic stimulation of wM1 evokes cortical activation in wS1 in the absence of whisker movement, and pharmacological inhibition of wM1 increases LF activity in wS1 during both quiet and AW, though it does not abolish the state change during active behavior (Zagha et al., 2013). The cellular pathway from wM1 activity to wS1 state change is not completely clear as axons from wM1 target many different subtypes of neurons in the superficial layers of wS1, including excitatory pyramidal cells, PV, SOM and VIP interneurons. However, it is known that the VIP interneurons, in particular, receive a strong drive from wM1 and in turn inhibit SOM interneurons leading to disinhibition of pyramidal cells (Lee et al., 2013).

In other cortical areas, the mechanisms of locomotor induced state change have been less studied. Increase in thalamic activity as well as involvement of cortical cholinergic inputs likely also participate in state change in V1 (Bennett et al., 2013; Erisken et al., 2014; Fu et al., 2014; Chen et al., 2015; Reimer et al., 2016). The involvement of the thalamic drive in Au1 is debated (Zhou et al., 2014) but cholinergic inputs as well as top-down input from secondary motor cortex (M2) may play a stronger role (Schneider et al., 2014; Nelson and Mooney, 2016; Reimer et al., 2016). Overall, neurons in the central/dorsal medial thalamic nuclei could play an important role in initiating and maintaining general cortical arousal (Gent et al., 2018; Mátyás et al., 2018). Other sources of modulatory inputs including noradrenergic inputs from the brainstem (Constantinople and Bruno, 2011; Polack et al., 2013; Fazlali et al., 2016; Reimer et al., 2016) or top-down inputs from higher-cortical areas (Zhang et al., 2014) also alter cortical activity and it is likely that multiple parallel pathways contribute to cortical activation with different involvement depending on the behavioral state (i.e., cortical activation during QW, whisking, locomotion) or context (spontaneous behavior vs. engagement in a task).

Recent studies have also pointed to possible circuits involved in the control of cortical states at a more local level. The cholinergic neurons from the basal forebrain innervate specific regions of the neocortex with little overlap—i.e., the cholinergic neurons that send axons to somatosensory cortex are not the same as the ones that project to the visual or the auditory cortex. Thus, cholinergic neurons can control regional cortical activation in a modality-selective manner (Zaborszky et al., 2015; Kim et al., 2016; Záborszky et al., 2018). Another circuit that could control cortical states locally comes from the thalamic reticular nucleus (nRT). The nRT is composed of GABAergic neurons that exert a strong inhibitory control onto thalamocortical neurons and is a key player in the control of the thalamo-cortical loop (Pinault, 2004; Fuentealba and Steriade, 2005). It has been shown recently that the nRT could play an important role in controlling cortical activation during arousal (Herrera et al., 2016). Importantly, localized activation of a small region of the nRT can produce local cortical deactivation (Lewis et al., 2015). These results are in line with the local state changes observed in the cortex of awake rats or mice (Vyazovskiy et al., 2011; Fernandez et al., 2017) and suggest that cortical states can be modulated both at a global and local level.

Thus, various cortical and subcortical structures that innervate S1 can trigger cortical state changes, but where is the earliest signature of activity in the brain that could possibly initiate the change in cortical state? Classical recording and stimulation studies identified brainstem nuclei as major drivers of cortical state both via direct cortical projections and indirect activation of subcortical nuclei. For example, the cholinergic neurons of the pedunculopontine tegmental nucleus and laterodorsal tegmental nucleus (PPT/LdT) are more active during states of vigilance characterized by cortical activation (wakefulness and paradoxical sleep; Sakai, 2012; Boucetta et al., 2014) and electrical stimulation of PPT and LdT can evoke strong cortical activation with suppression of slow-cortical activities (Steriade et al., 1993a). These neurons do not project directly to the cortex but densely innervate and activate different thalamic nuclei (Hirata and Castro-Alamancos, 2010), as well as histaminergic neurons in the posterior hypothalamus and cholinergic cells in the basal forebrain (Lin, 2000; Jones, 2005). In the same region, the noradrenergic neurons of the locus coeruleus display also tonic changes in neuronal activity correlated to the current state of vigilance, with highest activity during wakefulness (Jones, 2005). Moreover, these neurons show strong phasic activity in response to salient sensory stimuli (Aston-Jones and Cohen, 2005). These neurons provide a direct, widespread innervation of the neocortex and their activation can also generate broad cortical activation (Steriade et al., 1993a; Kim et al., 2016). Other neuronal populations in the brainstem might also be responsible for the initiation of spontaneous locomotor activity (Lee et al., 2014). Moreover, cortical state changes could be initiated indirectly via top-down inputs originating in the cortex. The medial prefrontal cortex (mPFC), for example, projects both to cholinergic neurons in the basal forebrain that innervate the neocortex (Golmayo et al., 2003; Vertes, 2004) and to the nRT (Wimmer et al., 2015; Phillips et al., 2016). Thus, most likely, a number of sources can initiate global and/or local cortical state changes. Future work should examine animals under many behavioral conditions to test the hypothesis that dedicated circuits are used under specific behavioral demands.

## Local and Long-Range Synchrony

An important aspect of brain activity for the encoding of information is the level of synchrony between neurons or population of neurons. Neuronal synchrony is thought to have important impact on neuronal processing at different spatial scales (Engel et al., 2001; Averbeck et al., 2006; Smith and Kohn, 2008). Local synchronization between small neuronal populations may allow for the formation of functional neuronal ensembles coding for similar aspect of a stimulus (Smith and Kohn, 2008; Ko et al., 2011). On the other hand, local desynchronization may enhance the information content of neuronal population activity (Beaman et al., 2017). At larger scale, long-range interareal synchronization between brain regions may underlie functional coupling of areas co-engaged in a given cognitive task (Engel and Singer, 2001; Varela et al., 2001; Buzsáki and Draguhn, 2004; Melloni et al., 2007).

**Figure 5:**
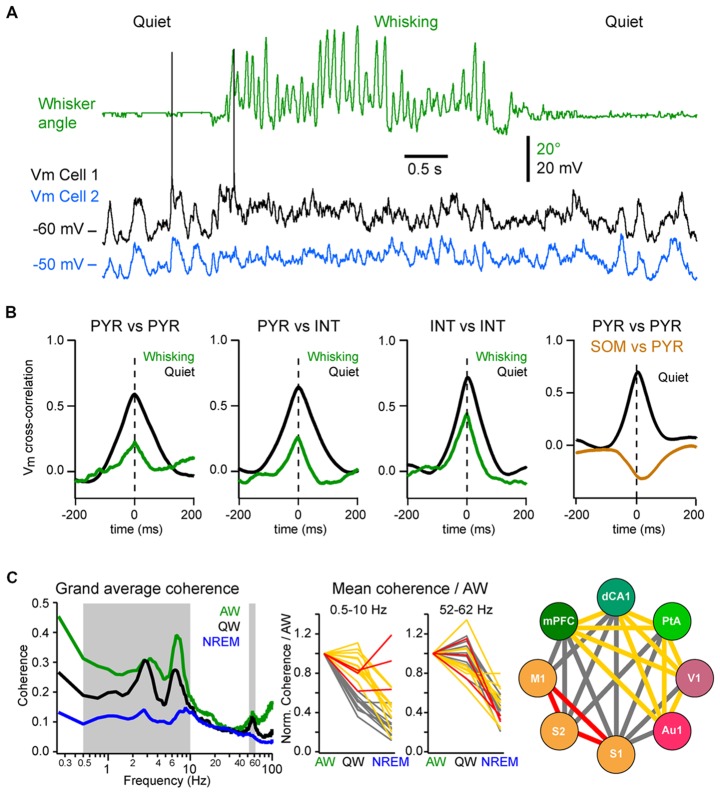
Local and long-range synchrony.** (A)** Neuronal synchrony can be assessed by the cross-correlation of the Vm of simultaneously recorded neurons in wS1. Adapted from Poulet and Petersen (2008) with permission from Springer Nature. **(B)** Motor activity (whisking) decreases the synchronization between nearby neurons. From left to right, mean Vm cross-correlations during QW (black) and whisking (green): between L2/3 excitatory (PYR) neurons; between PYR and GABAergic interneurons (INTs); between INTs; and between PYR and SOM expressing interneurons (SOM) during QW only. Note the antiphase-correlation of the Vm between SOM and PYR. Adapted from Gentet et al. (2010) with permission from Elsevier and Gentet et al. (2012) with permission from Springer Nature. **(C)** Long-range synchrony can be assessed by the measurement of the coherence between LFPs recorded simultaneously from different cortical areas. *Left*, the interareal coherence is overall maximal in the LF range during AW and is strongly reduced during non-rapid eye movement (NREM) sleep in awake and naturally sleeping mice. *Middle*, change in coherence in the LF (0.5–10 Hz) range relative to AW across areas reveals three groups of cortical areas: areas showing a maintenance of, or an increase in, the coherence during NREM sleep compared to QW (red); areas showing the strongest decrease of coherence between QW and NREM sleep (yellow) and areas showing the strongest decrease from AW to QW (gray). *Right*, change in coherence in the LF range reveals a functional organization of the cortical areas: somatosensory and motor areas that are directly synaptically connected maintain a high coherence during NREM sleep; the other areas maintain coherence throughout wakefulness but not during NREM sleep; the coherence between the two groups drops already during QW compared to AW. Adapted from Fernandez et al. (2017) with permission from Oxford University Press.

Neuronal synchrony is highly dynamic and is strongly affected by brain states (Harris and Thiele, 2011). During QW, the slow, high-amplitude Vm fluctuations of nearby excitatory cortical neurons are highly synchronous, both within the same layer (Figures 5A,B; Poulet and Petersen, 2008; Gentet et al., 2010; Arroyo et al., 2018) and across layers with deeper layer neurons depolarizing before superficial neurons in the same column (Zhao et al., 2016). Vm fluctuations are also highly synchronized between pairs of inhibitory interneurons and between excitatory cells and most interneurons, except for the SOM interneurons (Figure 5B; Gentet et al., 2010, 2012). This LF activity is also well synchronized across more distant cortical areas. The interareal coherence is high during QW and shows two prominent peaks in the delta (1–4 Hz) and Theta (4–10 Hz) frequency bands (Figure 5C; Fernandez et al., 2017). The long-range synchronization of cortical activity in the LF band—in particular in the delta band—during QW could reflect the coupling between the respiration and cortical activity, that appears to be particularly prominent in the ventral and mPFC and hippocampus (Nguyen Chi et al., 2016; Biskamp et al., 2017; Karalis and Sirota, 2018; Kőszeghy et al., 2018), but also extend to sensory areas (Ito et al., 2014; Rojas-Líbano et al., 2018) and could synchronize brain rhythms more globally (Karalis and Sirota, 2018; Rojas-Líbano et al., 2018; Tort et al., 2018). It should be noted however that although the local neuronal activity is highly correlated during high-amplitude, LF cortical activities, this is not always the case for long-range interactions. Indeed, during non-REM sleep, cortical LF activity increases throughout the cortex—especially the slow and delta-oscillations (0.5–5 Hz)—but the interareal coherence is overall strongly decreased in the LF band (Figure 5C; Fernandez et al., 2017). This is in contrast with the highly-synchronized slow-oscillation observed across cortical areas under anesthesia (Amzica and Steriade, 1995; Isomura et al., 2006; Volgushev et al., 2006; Busche et al., 2015) but is consistent with a more regional slow-wave activity (Chauvette et al., 2011; Nir et al., 2011; Busche et al., 2015) and a decrease in cortical functional connectivity during non-REM sleep (Massimini et al., 2005; Olcese et al., 2016; Fernandez et al., 2017). Nevertheless, highly synchronous slow-wave activity can occur during non-REM sleep between directly connected cortical areas (Miyamoto et al., 2016; Fernandez et al., 2017) or in other frequency bands (Le Van Quyen et al., 2016) that could play an important role in memory formation (Le Van Quyen et al., 2016; Miyamoto et al., 2016). Thus, the LF cortical activity observed during QW forms a very different local and global spatiotemporal pattern of neuronal synchrony compared to the slow-oscillation that characterizes non-REM sleep.

During AW, cortical activation is associated with a decrease in local neuronal synchrony. In the primary somatosensory cortex of awake mice, Vm fluctuations are less correlated between nearby excitatory and inhibitory neurons than during QW (Figures 5A,B; Poulet and Petersen, 2008; Gentet et al., 2010; Zhao et al., 2016). Similarly, locomotion has been found to decrease pairwise neuronal correlation in the mouse visual cortex (Erisken et al., 2014; Arroyo et al., 2018). However, despite a reduction in local Vm synchrony during movement, multisite LFP recordings have shown that the interareal coherence in the LF band increases during AW compared to QW, despite an overall decrease in the power in the LF band (Figure 5C; Fernandez et al., 2017). Interareal Vm coherence needs further investigation, however the “functional connectivity” of neurons in different cortical areas, as measured by the level of correlation of action potential timing between two neurons, also shows an overall maintenance during AW compared to QW, in sharp contrast with the overall decrease observed during non-REM sleep (Olcese et al., 2016). Long-range interareal coherence can even be further increased temporally when an animal is engaged in a task. In rats performing a whisker tactile discrimination task, high coherence in the theta band was found between wS1 and the dorsal hippocampus as the animal whisked to approach and palpate the texture to be discriminated (Grion et al., 2016). Thus, despite the fact that AW is characterized by global cortical activation and local neuronal desynchronization, long-range synchronization in the low- and gamma-frequency bands seems to correlate with high arousal and attentional state. Additional studies should investigate further how long-range synchronization is affected when animals are engaged in a task.

## Impact of Cortical States on Sensory Processing

Brain states are tightly correlated to the level of vigilance or attention and have a strong impact on sensory processing. Sensory processing also occurs in different behavioral contexts, with sensory stimuli that can be sensed passively during immobile behaviors or actively gathered by moving the sensors during exploratory behaviors. Moreover, certain behavioral conditions require acute attendance to behaviorally relevant sensory stimuli, whereas in other behaviorally conditions, the same stimuli might be ignored. Sensory processing is thus highly dependent on behavioral and motivational states as well as context and past experience. As discussed in the previous paragraphs, the strongest and most ubiquitous effect of cortical activation in sensory areas—whether it is induced by motor activity or some attentional process—is to suppress large amplitude, slow Vm fluctuations. One of the first and most obvious effects of cortical activation is therefore to reduce the background neuronal fluctuations that interfere with sensory driven activity. As a consequence, cortical activation often results in an increased reliability of the sensory evoked neuronal responses—i.e., decreased variability of the evoked response across trials—and increased signal to noise ratio (SNR; Hirata and Castro-Alamancos, 2011; Bennett et al., 2013; Polack et al., 2013; Zhou et al., 2014; Vinck et al., 2015; Meir et al., 2018; Neske and McCormick, 2018).

**Figure 6:**
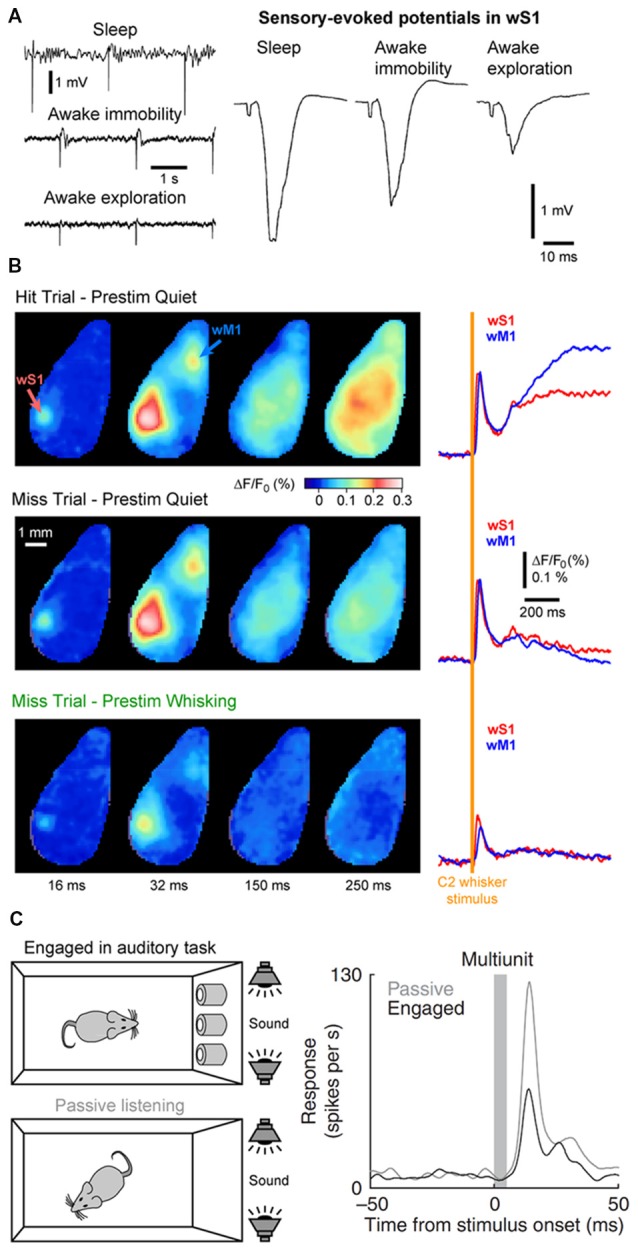
Cortical states and sensory processing.** (A)** Sensory evoked responses recorded in a freely moving rat change as function of behavioral states. *Left*, example LFP recordings from wS1 during periods of NREM sleep (sleep), QW (awake immobility) and AW (awake exploration). Electrical stimulation of the whisker pad are applied in the different states. *Right*, sensory-evoked potentials recorded in wS1 in response to the stimulation of the whisker pad in different behavioral states. Note the decrease of the evoked response during exploration compared to immobility. Adapted from Castro-Alamancos (2004) with permission from Elsevier. **(B)** The early sensory response in wS1 is mostly modulated by behavioral states but not by the behavioral output. Wide field images of the activity of the dorsal cortex using voltage sensitive dye (VSD) imaging in mice performing a whisker-based sensory detection task. From top to bottom: average response for successful trials (Hit) during which the mouse was not whisking before the whisker stimulus; unsuccessful trials (Miss) during which the mouse was not whisking before the whisker stimulus; and unsuccessful trials (Miss) during which the mouse was whisking before the whisker stimulus. Note the strong reduction of the early sensory evoked response, both in wS1 and wM1, when the stimulus occurs when the mouse is whisking (Prestim Whisking), whereas the early response is very similar whether the mouse responded (Hit) or not (Miss) when the stimulus occurs during QW (Prestim Quiet). Adapted from Kyriakatos et al. (2017) with permission from SPIE Digital Library. **(C)** The sensory-evoked response is strongly modulated by task engagement. Sensory evoked responses are measured in the Au1 of a rat that is engaged in an auditory-discrimination task or is passively exposed to the same auditory stimulus. The sensory-evoked response in Au1 (Multiunit) is markedly reduced when the rat is engaged in the task compared to passive listening. Adapted from Otazu et al. (2009) with permission from Springer Nature.

State changes not only affect the endogenous cortical activity but also the feedforward transfer of sensory information from the thalamus to the cortex. In the somatosensory system, during AW (or cortical activation) the cortical response evoked by a passive stimulus decreases in amplitude (Figure 6A; Fanselow and Nicolelis, 1999; Castro-Alamancos, 2002; Crochet and Petersen, 2006; Ferezou et al., 2006; Zhao et al., 2016). Moreover, cortical neurons respond with higher selectivity, less variability and show less adaptation to repetitive stimuli (Castro-Alamancos, 2002, 2004; Hentschke et al., 2006). Synaptic depression of thalamocortical synapses seems to be responsible, at least partly, for the decrease of the sensory evoked response and decrease of the adaptation during active waking (Castro-Alamancos and Oldford, 2002; Chung et al., 2002). As the firing rates of thalamocortical neurons increase during AW, sensory input is transmitted to the cortex via thalamocortical synapses already at a steady state of synaptic depression. Hence, the response to the first stimulus is smaller, however subsequent stimuli presented during AW do not lead to further synaptic depression and therefore have a lower overall variance than during QW (Castro-Alamancos and Oldford, 2002).

The sensory evoked response is also more spatially restricted during activated states. In anesthetized rats, cortical activation elicited by the stimulation of the brainstem reticular formation reduces the spreading of the sensory evoked response in wS1 (Castro-Alamancos, 2002). In awake mice, voltage sensitive dye (VSD) imaging allows for the measurement of the sensory evoked response throughout the dorsal cortex. When the mouse is still, the cortical response to a brief single-whisker deflection spreads rapidly from the principal-whisker barrel column to the wS2 and the wM1 before sometimes invading the entire dorsal cortex. In contrast, when the mouse is actively whisking, the response remains mostly restricted to wS1, wS2 and to a lesser extend wM1 (Ferezou et al., 2006, 2007). Together, these results suggest a reduced, but more precise and reliable representation of sensory input during AW.

However, the impact of motor activity on tactile sensory processing could be more complex. In the barrel cortex, L2/3 pyramidal neurons projecting to wS2 or wM1 are differentially affected by the state change. During QW, the neurons projecting to wM1 exhibit a stronger and faster response to a brief passive stimulus than wS2 projecting neurons. However, during whisking, the response of wM1 projecting neurons is reduced in amplitude whereas the response of wS2 projecting neurons is maintained. Furthermore, during active contact with an object, wS2 projecting neurons respond more reliably to each contact whereas the response of wM1 projecting neurons shows strong depression across successive contacts (Yamashita et al., 2013). Therefore, different behavioral states appear to recruit different neuronal pathways, with sensory signals to passive stimuli being better relayed to wM1 during QW and sensory signals gathered through active sensing being better relayed to wS2. The differential recruitment of these two pathways downstream of wS1 could have a functional relevance when rodents use their whiskers for different purposes such as object location or texture discrimination (Chen et al., 2013).

Interestingly, locomotor activity has different effect on sensory processing across sensory modalities. Similar to tactile processing, auditory evoked responses are also reduced in the Au1 during locomotion (Schneider et al., 2014; Zhou et al., 2014; McGinley et al., 2015a). However, in the V1, sensory responses are enhanced during locomotion (Niell and Stryker, 2010; Ayaz et al., 2013; Bennett et al., 2013; Polack et al., 2013; Erisken et al., 2014; Reimer et al., 2014; Vinck et al., 2015; Dipoppa et al., 2018; Neske and McCormick, 2018). While visual stimuli are typically longer than the brief stimuli used in somatosensory or auditory studies, one interpretation of this difference could be that different sensory modalities dominate depending on the behavioral state. Immobile, QW could be more adapted to the detection of small passive whisker deflections (Ollerenshaw et al., 2012; Kyriakatos et al., 2017) or sounds (McGinley et al., 2015a) to alert the animal to possible predators. It is probably particularly important for running rodents, on the other hand, to be particularly sensitive to moving visual stimuli. In good agreement mice show improved visual detection during locomotion (Bennett et al., 2013). Therefore, rodents may prioritized vision over audition during locomotion.

The impact of cortical states on the *perception* of sensory stimuli is a more challenging question to address. It requires the subject under investigation to be able to report whether a given sensory stimulus was detected during different cortical states. Over the last decade, a major effort has been made to design behavioral tasks for head-fixed rodents to tackle this question. In a simple task, water-restricted head-fixed mice report the detection of a target sensory stimulus—a brief whisker deflection—by licking a water spout to obtain a small drop of water as a reward (Miyashita and Feldman, 2013; Sachidhanandam et al., 2013; Yang et al., 2016). It is then possible to compare the cortical activity for trials in which the animal successfully reported the stimulus (Hit trials) and trials in which the animal failed to detect the stimulus (Miss trials). Data from wS1 recordings during this task has shown that the early sensory evoked response is not correlated to the subsequent performance (Hit vs. Miss) but is strongly modulated by the motor activity (Quiet vs. Whisking; Figure 6B; Sachidhanandam et al., 2013; Kyriakatos et al., 2017). Moreover, the cortical state (assessed by the spontaneous Vm fluctuations) preceding the whisker stimulus in wS1 was found to have little impact on the detection probability (Sachidhanandam et al., 2013). In contrast, both the cortical state and sensory processing are strongly modulated by task engagement, with an overall decrease in LF cortical activity and decrease in sensory evoked subthreshold response in mice engaged in the task compared to mice passively exposed to the stimulus (Sachidhanandam et al., 2013). Similar results have been found in the auditory cortex, where the sensory evoked spiking response of rats engaged in an auditory-discrimination task is markedly reduced compared to the response evoked in the same rats passively listening to the same auditory stimulus (Figure 6C; Otazu et al., 2009). Similarly, task engagement was found to decrease the sensory evoked response in most excitatory neurons in the mouse auditory cortex. The effect was mediated by a cholinergic-dependent increase in activity of layer 4 inhibitory interneurons (Kuchibhotla et al., 2017).

In the visual cortex of rodents (Pinto et al., 2013) or primates (Engel et al., 2016; Beaman et al., 2017), local cortical states have been found to correlate with performance, with a significant improvement of sensory detection or discrimination during desynchronized local states in V1. The reason for this difference across sensory modalities is unclear. It is possible that local brain states have different effects on sensory perception in different cortical areas, it could also be that the exact statistics of the sensory stimulus makes the task more or less sensitive to brain states—i.e., very salient sensory stimuli that are easy to detect or discriminate are probably equally perceived in synchronized or desynchronized states, whereas less salient or more complex stimuli might be better perceived in desynchronized than synchronized states (Bennett et al., 2013; Pinto et al., 2013). Non-linear cortical processing that selectively enhances small inputs during depolarized Vm states may participate to the better detection of less salient, weaker sensory stimuli in the desynchronized state (Reig et al., 2015; Ferrarese et al., 2018). Another possible confounding factor might be due to the interpretation of the Miss trials. They are often assumed to be the result of a failure of perception but could remain unreported because of a lack of motivation. Indeed, in sensory detection tasks in rodents, Miss trials often occurs when the animal is either very active or is disengaged from the task (McGinley et al., 2015a; Kyriakatos et al., 2017). Obviously, the brain states during high locomotor activity and disengagement from the task would be very different and pooling the Miss trials together might lead to a misconception of the impact of brain state on sensory perception (Jacobs et al., 2018). Recent studies using sensory decision-making tasks have pointed to an “optimal” brain state for sensory detection. Task performance is higher during immobile, quiet behavior with high arousal than during lower level of arousal or high motor activity (McGinley et al., 2015b; de Gee et al., 2018; Neske and McCormick, 2018; van Kempen et al., 2018). These changes in brain state can occur spontaneously during the task or reflect overall changes in task engagement (Ganea et al., 2018; Jacobs et al., 2018), Moreover, they are well correlated to pupil size and could relate partly to fluctuations in the activity of locus coeruleus noradrenergic neurons in the brainstem (de Gee et al., 2017, 2018).

Finally, brain states and sensory processing are likely to be modulated by other task-related parameters, such as behavioral context and experience. Behavioral context likely defines and enhances the processing of relevant sensory stimuli (Itskov et al., 2012; Lee et al., 2016). Moreover, learning is thought to enhance the processing of behaviorally relevant sensory stimuli while suppressing responses to irrelevant stimuli (Yamashita and Petersen, 2016; Schneider et al., 2018). Upon learning, the sensory processing for relevant stimuli could also extend to higher-order cortical areas, such as the mPFC and the dorsal hippocampus (Pinto and Dan, 2015; Otis et al., 2017; Le Merre et al., 2018). The modulation of brain states and sensory processing by attention, engagement or learning could rely on the tonic and phasic release of acetylcholine and/or noradrenaline that could signal shift in attention, reward or saliency (Shulz et al., 2000; Ego-Stengel et al., 2001, 2002; Fazlali et al., 2016; Vazey et al., 2018). In future studies, it will be important to design specific behavioral tasks that directly address the role of different behavioral factors (perception, attention, engagement, arousal) on brain states and sensory processing.

## Conclusion

In summary, studies in awake animals have shown that spontaneous, internally-generated input is the dominant component of the Vm activity of a cortical neuron. It forms distinct patterns of activity, termed cortical states, during different states of behavior and arousal and has a clear influence both on the synchrony of cortical neuron activity as well as the processing of sensory input. While the circuit mechanisms of global changes in cortical state have begun to be understood in the mouse, future studies should now address the control of fine spatial scale changes of cortical state observed in cortex wide recordings. To better assess the influence of cortical states on perception and movement, behavioral tasks must now be designed to better assess the levels of arousal and attention possibly using less salient, near threshold stimuli. The diversity and prevalence of cortical states in awake animals suggests that, rather than being treated as a source of noise to be averaged out, they should be considered as a rich and fundamental aspect of cortical processing.

## Author Contributions

SC and JP wrote the manuscript. All authors contributed to manuscript revision, read and approved the submitted version.

## Conflict of Interest Statement

The authors declare that the research was conducted in the absence of any commercial or financial relationships that could be construed as a potential conflict of interest.

## Footnotes

^1^In humans, as well as in many mammals, non-REM sleep is divided into 2–4 stages depending on the dominant oscillatory activities in the EEG, from sleep spindles (10–15 Hz), to delta (1.5–5 Hz) and slow-oscillation (0.1–1.5 Hz).

^2^In pyramidal cells, the state change often leads to an apparent contradictory effect with a more depolarized mean Vm but a decrease in firing rate. The explanation comes from the decrease in the amplitude of the fluctuations of the Vm (the SD of the Vm) that accompanied the mean depolarization. As the result, although the Vm is sitting closer to the threshold for spike initiation it is in fact less likely to cross it.
